# Supplementary material for: Voltage sensor conformations induced by LQTS-associated mutations in hERG potassium channels
Source: Nat Commun. 2025 Aug 3;16:7126. doi: 10.1038/s41467-025-62472-9 (PMC12318091; doi:10.1038/s41467-025-62472-9)
Supplement: Supplementary file 2 — Description of Additional Supplementary Files [file 41467_2025_62472_MOESM2_ESM.pdf]

Description of Additional Supplementary Files

Supplementary Movie 1: Steered MD simulations of the transmembrane domains of the wild-type hERG channel, showing the upward movement of the S4 helix.

Supplementary Movie 2: Steered MD simulations of the transmembrane domains of hERG-R531Q channel, showing the upward movement of the S4 helix.

Supplementary Movie 3: Steered MD simulations of the transmembrane domains of hERG-R528Q channel,
showing the upward movement of the S4 helix.
